# Histone Deacetylase Activity Selectively Regulates Notch-Mediated Smooth Muscle Differentiation in Human Vascular Cells

**DOI:** 10.1161/JAHA.112.000901

**Published:** 2012-06-22

**Authors:** Yuefeng Tang, Joshua M. Boucher, Lucy Liaw

**Affiliations:** Center for Molecular Medicine, Maine Medical Center Research InstituteScarborough, ME

**Keywords:** signal transduction, muscle, smooth, vasculature, cell differentiation

## Abstract

**Background:**

Histone deacetylases (HDACs) modify smooth muscle cell (SMC) proliferation and affect neointimal lesion formation by regulating cell cycle progression. HDACs might also regulate SMC differentiation, although this is not as well characterized.

**Methods and Results:**

Notch signaling activates SMC contractile markers and the differentiated phenotype in human aortic SMCs. Using this model, we found that HDAC inhibition antagonized the ability of Notch to increase levels of smooth muscle α-actin, calponin1, smooth muscle 22α, and smooth muscle myosin heavy chain. However, inhibition of HDAC activity did not suppress Notch activation of the HRT target genes. In fact, HDAC inhibition increased activation of the canonical C-promoter binding factor-1 (CBF-1)–mediated Notch pathway, which activates HRT transcription. Although CBF-1–mediated Notch signaling was increased by HDAC inhibition in human SMCs and in a C3H10T1/2 model, SMC differentiation was inhibited in both cases. Further characterization of downstream Notch signaling pathways showed activation of the c-Jun N-terminal kinase, p38 mitogen-activated protein kinase, and PI3K/Akt pathways. The activation of these pathways was sensitive to HDAC inhibition and was positively correlated with the differentiated phenotype.

**Conclusions:**

Our studies define novel signaling pathways downstream of Notch signaling in human SMCs. In addition to the canonical CBF-1 pathway, Notch stimulates c-Jun N-terminal kinase, mitogen-activated protein kinase, and PI3K cascades. Both canonical and noncanonical pathways downstream of Notch promote a differentiated, contractile phenotype in SMCs. Although CBF-1–mediated Notch signaling is not suppressed by HDAC inhibition, HDAC activity is required for Notch differentiation signals through mitogen-activated protein kinase and PI3K pathways in SMCs. **(J Am Heart Assoc. 2012;1:e000901 doi: 10.1161/JAHA.112.000901)**

## Introduction

Smooth muscle cells (SMCs) have a unique ability to modify their contractile phenotype to a transitional state during the pathogenesis of vascular diseases. This transitional state can include alterations in morphology, gene expression, contraction, and proliferation. Typically, reduction in contractile proteins is concurrent with entry into the cell cycle and increased migratory ability. Several signaling pathways are well-characterized regulators of SMC phenotype, and many function in a cooperative manner.^1–4^ Our laboratory has focused on the Notch signaling pathway as a critical regulator of SMC phenotype. Notch signaling promotes SMC differentiation via direct activation of contractile gene transcription as well as regulation of SMC microRNAs,^5–9^ and mutations in the Notch pathway are implicated in human vascular pathologies.^10–12^

There is interest in epigenetic modifications that might potentially impact human cardiovascular diseases.^13^ In particular, histone acetylation and deacetylation are major determinants of chromatin structure and gene transcription. Enzymes of the histone deacetylase (HDAC) family generally inhibit transcription. A major mechanism of tumor suppressor gene silencing in cancers by HDACs has led to the use of HDACinhibitors as anticancer therapeutics.^14,15^ Several lines of evidence implicate HDACs as a target for regulation of SMC phenotype. Inhibition of HDAC activity can alter SMC proliferation.^16–19^ Although there is some discrepancy, most in vivo studies show that HDAC inhibition suppresses neointimal lesion formation,^18,20^ which suggests a potential therapeutic target for cardiovascular diseases. Indeed, HDAC inhibitors prevent cardiac hypertrophy, heart failure, and hypertension in rodent models.^21–24^

A few studies have addressed HDAC regulation of SMC differentiation. Suppression of SMC markers by platelet-derived growth factor-BB is mediated partially by recruitment of HDACs to contractile gene promoters.^25^ In addition, suppression of SMC differentiation by oxidized phospholipids was mediated by Krüppel-like factor 4, E twenty-six–like transcription factor 1 (Elk1), and HDAC activity on genes, including smooth muscle α-actin (SM actin).^26^ Transforming growth factor β (TGFβ)–mediated induction of smooth muscle 22α (SM22α) expression was concurrent with hyperacetylation of this locus, and HDAC inhibitors enhanced TGFβ promotion of SM22α transcription.^27^ Recently, a link of HDACs to Notch signaling was discovered in studies that conditionally deleted HDAC3 in neural crest cells, which give rise to subpopulations of arterial SMCs.^28^ Loss of HDAC3 impaired development of arterial SMCs in the aortic arch, and this defect was concomitant with reduced expression of Jagged1. These data suggest that epigenetic regulation of SMC precursors can function upstream of Notch signaling. HDAC activity is also associated with a regulatory function in Notch signaling in other cells,^29–32^ but effects on Notch-mediated SMC differentiation are unknown.

In the present study, we tested the idea that SMC differentiation mediated by the Notch pathway is regulated by HDAC activity. Our studies in human primary SMCs with Notch as a differentiation factor revealed distinct phenotypes compared to rat SMC differentiation induced by TGFβ.^27^ Inhibition of HDAC activity in human SMCs downregulated Fbw7 and increased Notch1 protein. However, HDAC inhibition suppressed Notch-mediated SMC differentiation, but this was not due to inhibition of canonical C-promoter binding factor-1 (CBF-1)–mediated signaling. Other pathways activated by Notch in human SMCs include PI3K/Akt, c-Jun N-terminal kinase (JNK), and p38 mitogen-activated protein kinase (MAPK) signaling, all of which were suppressed by HDAC inhibition. Our studies show that regulators of SMC differentiation that might use multiple signal mediators can be selectively sensitive to epigenetic modifiers of gene expression.

## Methods

### Cell Culture

Human aortic SMCs (Cambrex, Walkersville, MD) were maintained in SmGM2 medium and were used between passages 4 and 7. Murine C3H10T1/2 fibroblasts were cultured in minimum essential medium with Earle's salts containing 10% fetal bovine serum, L-glutamine (2 mmol/L), 1% nonessential amino acids, 100 IU/mL penicillin, and 100 μg/mL streptomycin.

### Reagents

Trichostatin A (TSA) and dimethyl sulphoxide were from Sigma Aldrich (St. Louis, MO), and the HDAC class I inhibitor MS-275 was from Selleck (Houston, TX). Kinase inhibitors U0126, SB203580, SP600125, and LY294002 and the protease inhibitor cocktail were obtained from EMD Biosciences (Madison, WI).

### Quantitative Reverse-Transcription Polymerase Chain Reaction and Immunoblotting

Total RNA was extracted with Tri-reagent (Sigma), treated with RNase-free DNase I (Promega, Madison, WI), and reverse-transcribed with qScript cDNA SuperMix (Quanta Biosciences, Gaithersburg, MD). Quantitative reverse-transcription polymerase chain reaction (RT-PCR) was performed in the iCycler (Bio-Rad, Hercules, CA) using SYBR Green (Bio-Rad) with 20 ng cDNA as template, in triplicate. Threshold cycle numbers were calculated at log phase of amplification and normalized to cyclophilin. Soluble cell extracts were prepared, and aliquots containing 20 μg of total protein were separated by sodium dodecyl sulfate polyacrylamide gel electrophoresis and transferred to polyvinylidene fluoride membranes. The membranes were probed with antibodies against corresponding proteins, as described.^5^

### Gene Silencing With siRNA

Transfections were performed with the human aortic SMC–optimized Amaxa nucleofector system (Lonza, Walkersville, MD). Knockdown of CBF-1 was accomplished using 180 pmol siRNA or scrambled All Star Control (Qiagen, Valencia, CA) with 1×10^6^ cells per reaction, and transfection was done with the V-025 program. Cells were plated for 24 hours before transduction adenoviral constructs. Knockdown of serum response factor (SRF) was accomplished using 125 pmol siRNA or scrambled All Star Control with 5×10^5^ cells, and electroporation was done with the U-025 program. Cells were cultured for 48 hours before transduction with adenoviral constructs for subsequent analysis.

### Transient Transfections and Luciferase Assay

Human aortic SMCs were plated at 40 000 cells per well in a 12-well plate and were transduced with adenovirus (100 TCID_50_ virus particles per cell), 0.25 μg reporter plasmid, 0.75 μL Gene Juice (Invitrogen), and 25 ng of Renilla luciferase plasmid per well. Two days after transfection, cells were collected for luciferase assay, as described.^6^ All experiments were repeated ≥3 times, and representative results are shown. The SM actin promoter reporter constructs p125 and pA125^33^ were generously provided by Gary K. Owens, University of Virginia.

### Statistical Analysis

Statistical analyses to test differences between groups were performed with analysis of variance (ANOVA) in conjunction with the Tukey range test to determine significant differences at *P*<0.05. Data are presented as mean ± standard error of the mean (SEM). All experiments were performed independently ≥3 times. For quantitative RT-PCR analysis, each experimental group was tested in duplicate for each trial, and for luciferase assays, each group was tested in triplicate for each trial.

## Results

### HDAC Inhibition Abrogates Notch-Induced SMC Differentiation

**Figure 1.**
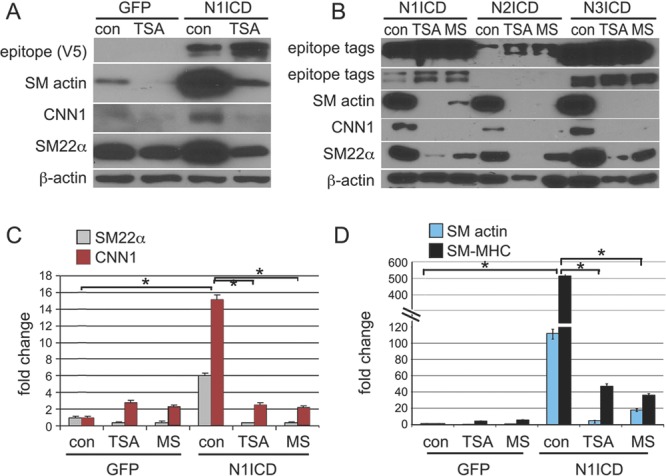
HDAC inhibition represses Notch-mediated SMC differentiation. A, Primary human aortic SMCs were transduced with Notch1ICD (N1ICD) or green fluorescent protein (GFP) and then were treated with the HDAC inhibitor TSA or control vehicle dimethyl sulphoxide (DMSO) (con) for 48 h before collection of cell lysates for immunoblot analysis. Expression of N1ICD was confirmed using the V5 epitope tag. Smooth muscle α-actin (SM actin), calponin1 (CNN1), and SM22α were analyzed and compared to levels of β-actin. B, Notch1ICD, Notch2ICD, and Notch3ICD were transduced into human aortic SMCs, which were then treated with HDAC inhibitors TSA or MS-275 or with vehicle DMSO (con). The top 2 rows are different exposures of the same blot to detect the epitope tags on the NICD constructs. Longer (top row) and shorter (second row) exposures are shown because the level of N2ICD expression was lower than that of N1ICD and N3ICD. SMC markers were analyzed and were similarly induced by activation of each Notch receptor. Both TSA and MS-275 significantly suppressed the induction of SMC proteins by Notch activation. C and D, Total RNA was collected under the same conditions, and transcripts for SMC markers were quantitatively measured in comparison to control SMCs without HDAC inhibitor treatment. Data are presented as mean±SEM and were analyzed for statistical significance by ANOVA / Tukey test. The asterisks indicate *P*<0.05. N1ICD significantly increased transcripts for all SMC markers tested, and TSA and MS significantly suppressed this induction.

It was shown previously that HDAC inhibition suppresses TGFβ-induced fibroblast–myofibroblast differentiation^34,35^ and regulates SMC proliferation and migration.^13,18^ We have characterized Notch signaling as a strong inducer of the contractile phenotype in human SMCs.^5,6^ No previous work has addressed the interaction of Notch signaling and HDAC activity in human SMCs. Therefore, our study was designed to test the effect of HDAC inhibition on Notch-induced SMC differentiation using transient expression of the constitutively active Notch intracellular domain (ICD) in the presence or absence of the HDAC inhibitor TSA for 2 days. Consistent with our previous report,^6^ human SMCs with activated Notch1 signaling dramatically increased levels of SM actin, calponin1 (CNN1), and SM22α (Figure 1A). Inhibition of HDAC activity reduced basal and Notch-induced activation of all differentiation markers (Figure 1A). We previously showed that human SMCs also express Notch2 and Notch3, which also promote a contractile phenotype when activated.^5^ To determine if HDAC inhibition also affected signaling downstream of different Notch receptor activation, cells were transduced with Notch1ICD, Notch2ICD, or Notch3ICD and then treated with TSA or MS-275 (entinostat), an HDAC class I–specific inhibitor. Inhibition of HDAC activity also repressed Notch2ICD- and Notch3ICD-mediated induction of SM actin, SM22α, and calponin1 (Figure 1B). Quantitative analysis of transcript levels determined that the effects of HDAC inhibition also were seen at the mRNA level. Induction of SM22α and calponin1 transcript was significantly suppressed in the presence of either HDAC inhibitor (Figure 1C), as were transcripts for SM actin and smooth muscle myosin heavy chain (Figure 1D). These data show that multiple SMC differentiation markers are inhibited by HDAC inhibition, with similar effects downstream of Notch1, Notch2, and Notch3 signaling.

### TSA Does Not Suppress the Notch/CBF-1 Pathway

**Figure 2.**
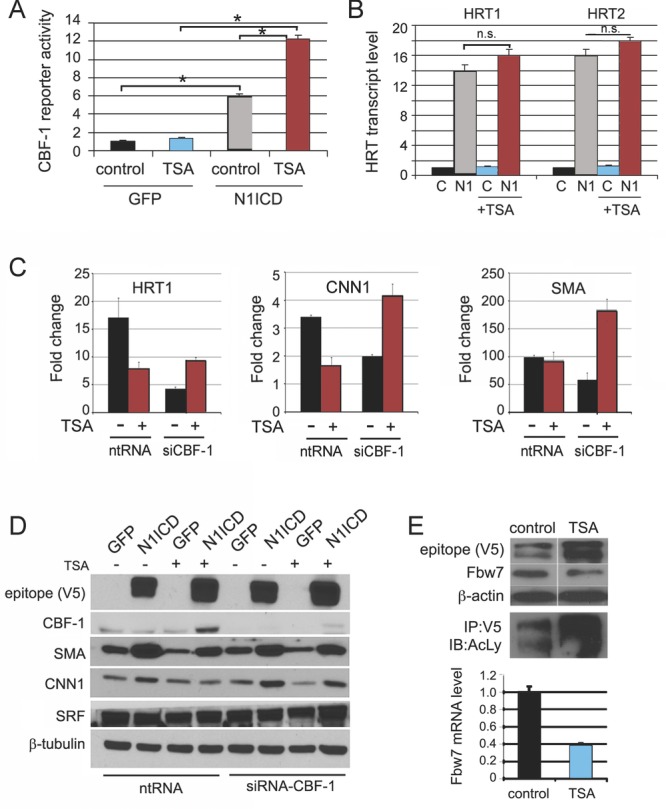
HDAC inhibition enhances Notch signaling and downregulates Fbw7. A, Primary human SMCs were transduced with green fluorescent protein (GFP) or Notch1ICD (N1ICD) and a CBF-1 luciferase reporter construct and were treated with TSA or control dimethyl sulphoxide (DMSO) for 48 h before analysis. Shown is normalized luciferase (mean±SEM). ANOVA / Tukey test was used for statistical analysis, and asterisks indicate *P*<0.05. Expression of N1ICD significantly increased reporter activity, even in the presence of TSA, and TSA further enhanced CBF-1 reporter activity in the presence of activated Notch signaling. B, GFP- or N1ICD (N1)–transduced SMCs were treated with TSA or control vehicle DMSO (C) for 48 h, and total RNA was collected for quantitative RT-PCR to measure HRT transcripts. Data were statistically analyzed by ANOVA / Tukey test, and HRT1 and HRT2 transcripts were significantly elevated with N1ICD or N1ICD+TSA compared to respective controls. There was no significant difference (n.s.) in N1ICD induction of HRT when TSA was included. C, CBF-1 was suppressed using specific siCBF-1 compared to nontargeting control (ntRNA), and quantitative RT-PCR was used to measure HRT1, calponin1 (CNN1), and SM actin (SMA) mRNA in the absence or presence of TSA. D, Protein lysates were collected from cells under the same conditions as in C and were used for immunoblot as indicated. E, N1ICD-transduced SMCs were treated with TSA or control vehicle DMSO for 48 h before analysis by immunoblot or quantitative RT-PCR to detect Fbw7 protein and transcript, respectively. Under the same conditions, 10% of total cell lysates were immunoprecipitated with anti-V5 and immunoblotted with an antibody recognizing acetylated lysine (AcLy).

To explore the mechanism of the inhibitory effect of TSA on Notch-mediated SMC differentiation, we examined whether TSA exerts its effects by antagonizing the Notch/CBF-1–dependent signaling pathway, which is central in promoting the SMC contractile phenotype.^5,6,36^ To assess this, CBF-1

luciferase reporter assays were performed. Although TSA treatment did not affect basal CBF-1 reporter activity, TSA enhanced Notch1ICD activation of CBF-1 activity (Figure 2A), which is consistent with studies in other cells.^29–32^ In addition, we quantified mRNA for HRT1 and HRT2, which are activated by NotchICD/CBF-1 complexes. TSA had no effect on the ability of Notch to activate these targets (Figure 2B). Thus, suppression of HDAC activity affects Notch targets in a gene-specific manner and does not appear to suppress Notch signaling via the canonical CBF-1 pathway. To further examine this pathway, CBF-1 was silenced in SMCs with siRNA, followed by activation of Notch1 signaling (Figure 2C). As expected, silencing CBF-1 protein decreased the ability of Notch1ICD to activate HRT1. Loss of CBF-1 also suppressed Notch induction of calponin and SM actin transcripts, but at only <50% reduction, which suggests that Notch is also activating alternative pathways leading to induction of SMC markers. Loss of CBF-1 with Notch1ICD expression led to a significant increase in calponin1 and SM actin transcripts with TSA treatment, which suggests that TSA activity is altered in the absence of CBF-1. Immunoblot analysis confirmed that suppression of CBF-1 protein did not completely block Notch1ICD-induced SM actin or calponin1 induction (Figure 2D). In addition, because SRF activity plays a dominant role in SMC differentiation, we analyzed SRF protein levels with Notch activation, TSA, and silenced CBF-1. The level of SRF did not change significantly under any condition (Figure 2D).

We also observed an apparent increase in transfected Notch1ICD protein with TSA treatment and tested whether HDAC inhibition affected Notch protein levels. The levels of Notch1 transcript were unchanged (data not shown), but there was a consistent increase in Notch1ICD protein after HDAC inhibition (Figure 2E), which suggests regulation of protein degradation. Fbw7 is an F-box protein that facilitates the ubiquitination and degradation of Notch receptors.^37–40^ Thus, we assessed whether TSA regulates Fbw7 expression. The results indicated that TSA represses Fbw7 expression at both the protein and the mRNA levels (Figure 2E). These data suggest that TSA enhances Notch signaling activity, at least in part by stabilizing Notch1ICD via the downregulation of Fbw7 in SMCs. The stabilization of Notch1ICD was consistent with increased acetylation. Cell lysates from control or TSA-treated cells were immunoprecipitated with anti-V5 (Notch1ICD epitope tag) and were immunoblotted with an antibody to acetylated lysines (Figure 2E, bottom blot). Increased acetylated Notch1ICD was associated with TSA treatment and is consistent with prior reports of acetylation-induced Notch1ICD stabilization.^41^

### The PI3K/Akt Pathway Is Required for Notch- Mediated SMC Differentiation

**Figure 3.**
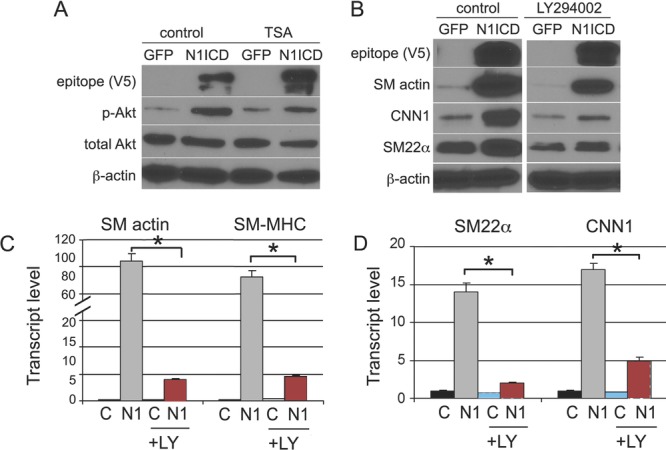
Notch activity requires PI3K/Akt signaling, which is sensitive to HDAC inhibition. A, Primary human SMCs were transduced with Notch1ICD (N1ICD) or green fluorescent protein (GFP) and then were treated with TSA or control vehicle dimethyl sulphoxide (DMSO) for 48 h before analysis. Cell lysates were collected for immunoblot to examine the activity of PI3K/Akt measured by p-Akt. B, N1ICD- or GFP-transduced SMCs were treated with the PI3K/Akt inhibitor LY294002 or DMSO control for 48 h before analysis. C and D, GFP- or N1ICD (N1)–transduced SMCs were treated with LY294002 (LY) or control vehicle (C) for 48 h, and total RNA was collected for expression analysis by quantitative RT-PCR for SMA and smooth muscle myosin heavy chain (SM-MHC) (C) and for SM22α and CNN1 (D). Graphed are fold changes compared with control SMCs without LY294002 treatment. Data are presented as mean±SEM and were statistically analyzed by ANOVA / Tukey test. Asterisks indicate *P*<0.05; LY294002 treatment significantly suppressed induction of all SMC marker genes by Notch activation.

Although HDAC inhibitors abrogate Notch-stimulated SMC differentiation, they also enhanced the NotchICD/CBF-1 pathway. This prompted us to assess whether HDAC inhibitors impede other pathways activated downstream of Notch signaling. It is known that the PI3K/Akt pathway is important for SMC differentiation,^42–44^ but Notch activation of this pathway is not well studied in SMCs. We first examined the extent to which Notch activation enhances PI3K/Akt activation. After expression of Notch1ICD, there was a significant increase in phosphorylated Akt (pAKT; Figure 3A), which is partially inhibited by HDAC inhibition. Using the PI3K inhibitor LY294002, we found that PI3K activity is indeed required for Notch stimulation of SMC markers, particularly protein accumulation of calponin1 and SM22α (Figure 3B). Notch induction of SM actin, smooth muscle myosin heavy chain, SM22α, and CNN1 transcripts was blocked by inhibition of PI3K activity (Figure 3C through 3D). These data show that multiple pathways, including PI3K/Akt signaling, are required for the full extent of Notch-mediated SMC differentiation.

### JNK and p38 MAPK Contribute to Notch1ICD-Mediated SMC Differentiation

**Figure 4.**
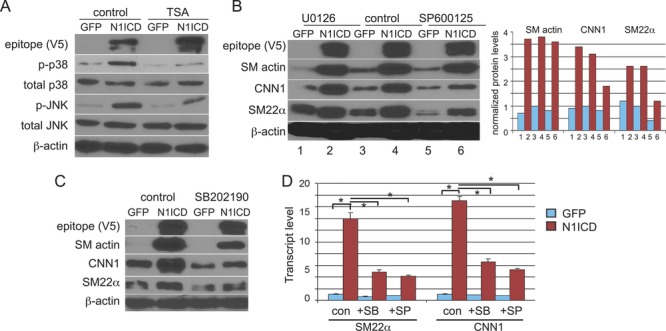
The MAPK pathway is involved in Notch-mediated SMC differentiation. A, Primary human SMCs were transduced with green fluorescent protein (GFP) or Notch1ICD (N1ICD) and were treated with TSA or control vehicle dimethyl sulphoxide (DMSO) for 48 h before analysis. Phosphorylated and total forms of p38 and JNK were measured. B, GFP- or N1ICD-transduced SMCs were treated with the MEK/Erk inhibitor U0126, JNK inhibitor SP600125, or control DMSO for 48 h before analysis by immunoblot for SMC markers. Protein levels are quantified in the graph on right. C, GFP- or Notch1ICD-transduced SMCs were treated with the p38 inhibitor SB202190 or control vehicle for 48 h before immunoblot analysis. D, GFP- or N1ICD-transduced SMCs were treated with the JNK inhibitor SP600125 (SP), the p38 inhibitor SB202190 (SB), or control vehicle DMSO (con) for 48 h, and total RNA was collected for expression analysis by quantitative RT-PCR for SM22α and CNN1. Data are presented as fold change compared to SMCs with control treatment. Data are presented as mean±SEM and were statistically analyzed by ANOVA / Tukey test. Asterisks indicate *P*<0.05; both JNK and p38 inhibitors significantly suppressed N1ICD induction of these genes.

Multiple signaling pathways are activated during SMC differentiation by other inducers, such as TGFβ1.^45–47^ We previously reported that activation of Notch signaling enhances TGFβ1 responsiveness in vascular SMCs,^5^ so we tested whether TGFβ1 pathways also were affected by Notch signaling. A pathway of interest is the MAPK pathway, including MEK/Erk, JNK, and p38. After Notch activation in SMCs, we observed significantly increased levels of the phosphorylated forms of JNK (p-JNK) and p38 (p-p38) but not Erk1/2 (Figure 4A and not shown). The inhibition of HDACs by TSA repressed Notch-induced JNK and p38 activation (Figure 4A). To further define the requirement of MAPKs in Notch activity in SMCs, we utilized inhibitors targeting MEK/Erk (U0126),^48^ JNK (SP600125),^49^ and p38 (SB201090).^50^ The inhibition of p38 and JNK pathways repressed Notch-mediated calponin1 and SM22α induction at both the mRNA and protein levels (Figure 4B through 4D). Also, inhibition of p38, but not JNK, moderately decreased Notch-mediated SM actin induction (Figure 4C). Erk signals are not required for Notch induction of SMC differentiation (Figure 4B) because Erk1/2 are not activated by Notch signaling, and U0126 did not change the response to Notch activation (Figure 4B). These data support the idea that HDAC inhibition represses Notch1ICD-mediated SMC differentiation via suppression of the p38 and JNK pathways.

### SRF Is Required for NotchICD-Induced SMC Differentiation

**Figure 5.**
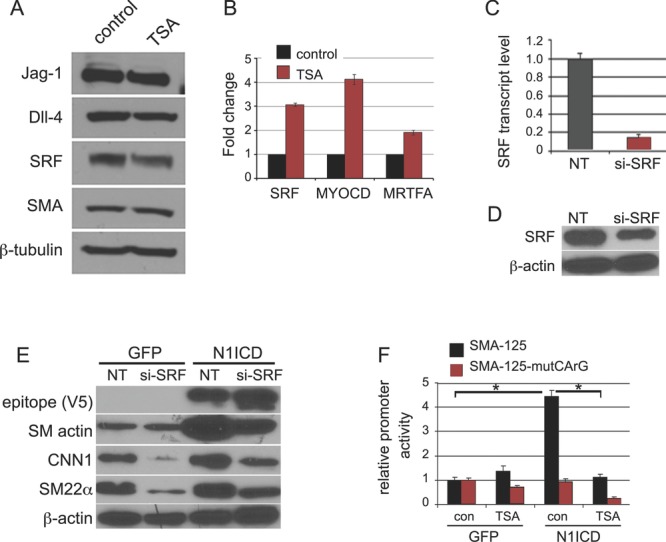
SRF is required for Notch-mediated SMC differentiation. A and B, Primary human SMCs were treated with TSA or vehicle control dimethyl sulphoxide (DMSO) for 48 h before Western blotting for Jag-1, Dll-4, SRF, and SMA protein levels (A) or quantitative RT-PCR analysis for SRF, myocardin (MYOCD), and MRTFA transcript levels (B). Data are presented as fold change as compared to SMCs treated with DMSO control. Human SMCs were transduced with a nontargeting siRNA (NT) or with siRNA targeted against SRF (si-SRF) for 4 days and were collected to examine the efficiency of knockdown by quantitative RT-PCR (C) and immunoblot (D). E, NT- or siSRF-transduced SMCs were infected with green fluorescent protein (GFP) or Notch1ICD (N1ICD) for 3 days and were collected for analysis of SMC markers. F, SMCs were transduced with GFP or N1ICD with the SM actin promoter reporter construct (SMA-125) or the construct with the mutant CArG box (SMA-125-mutCArG). Cells were treated with TSA or control vehicle DMSO (con) before analysis. Data are presented as fold change compared to SMCs with control DMSO treatment. Data are presented as mean±SD, and asterisks indicate *P*<0.05. SMA-125 activity was significantly increased with N1ICD compared to GFP, and this activity was significantly reduced with TSA.

One possible mechanism of action of HDAC inhibition is to alter the expression levels either of components of Notch signaling or of other factors that control smooth muscle differentiation. Although we found that TSA seemed to stabilize transduced Notch1ICD protein (Figure 2E), levels of the Notch ligands Jagged-1 and Dll-4 were not altered by TSA treatment (Figure 5A). SRF and its coactivators, myocardin and members of the myocardin-related transcription factor (MRTF) family, are important regulators of SMC phenotype.^51,52^ In addition, Notch signaling was reported to repress myocardin-regulated SMC differentiation via the Notch target gene HRT2.^53,54^ Thus, we addressed the interaction of SRF with Notch-mediated SMC differentiation. First, we assayed SRF protein levels under a variety of conditions, with TSA (Figure 2E and Figure 5A), with silenced CBF-1 (Figure 2E), and with activated Notch1 signaling^9^ (Figure 2E), and we found no evidence of changes in SRF protein levels. Because no suitable antibodies are available to detect myocardin and MRTFA by immunoblot, we performed quantitative RT-PCR to detect mRNA levels (Figure 5B). We observed a slight increase in the steady-state transcript levels for SRF, myocardin, and MRTFA. Although the changes in SRF mRNA did not translate into changes in protein level, we cannot exclude the possibility of increased levels of SRF cofactors. Therefore, we used siRNA to suppress SRF activity. SRF knockdown was confirmed by quantitative RT-PCR and immunoblotting (Figure 5C and 5D). SRF transcript was reduced by ≈80%, leading to significantly decreased SRF protein. In cells with reduced SRF, there was a dramatic decrease in basal SMC marker expression and the induction after Notch activation (Figure 5E). Thus, endogenous SRF contributes significantly to the differentiated phenotype of SMCs, but SRF is not the target of HDAC inhibition in these cells.

### The CArG Box Is Required for Notch1ICD-Mediated SM Actin Promoter Activity

The SM actin gene has a well-characterized promoter, and its transcription is sensitive to both SRF/myocardin and Notch signaling. To analyze whether TSA affects SM actin promoter activity, we used a luciferase promoter reporter (p125, SMA-125).^33^ As expected, activation of Notch1 significantly enhanced SM actin promoter activity, and this was blocked by HDAC inhibition (Figure 5F). To investigate the role of the SRF-responsive CArG box in Notch transcriptional activity, a CArG mutant (−62) p125 reporter plasmid (pA 125, SMA-125-mutCArG) was used.^33^ SMCs were transduced with green fluorescent protein, Notch1ICD, and SMA-125-mutCArG and then were treated with TSA or vehicle for 48 hours before luciferase assay. Activation of Notch was not able to promote transcription of the CArG mutant SM actin promoter (Figure 5F), consistent with the requirement of SRF activity for Notch-mediated differentiation.

### HDAC Inhibition Repressed Notch1ICD-Mediated SMC Differentiation From 10T1/2 Cells

**Figure 6.**
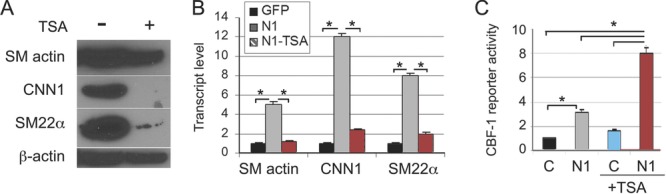
HDAC inhibition blocks Notch-mediated SMC differentiation in C3H10T1/2 progenitor cells. A and B, C3H10T1/2 cells were transduced with green fluorescent protein (GFP) or Notch1ICD (N1ICD) and were treated with TSA (+) or control (−) for 4 days before collection for immunoblot (A) or quantitative RT-PCR. C, C3H10T1/2 cells were transduced with the CBF-1 luciferase reporter construct and GFP control (C) or Notch1ICD (N1) and then were treated with TSA for 2 days. Graphed are fold changes compared to SMCs with control dimethyl sulphoxide (DMSO) treatment. Data are presented as mean±SEM, with asterisks indicating *P*<0.05.

We showed that Notch-mediated activation of several SMC markers is sensitive to HDAC inhibition in human primary SMCs. To test whether this mechanism is conserved in the differentiation of progenitor cells into SMCs, we used the C3H10T1/2 model, a murine embryonic mesenchymal precursor line that previously has been characterized to respond to Notch signaling by differentiation into the SMC lineage.^36^ To test the effects of TSA on this differentiation, we transduced cells with Notch1ICD in the absence or presence of TSA (Figure 6A). Similar to human SMCs, HDAC inhibition in C3H10T1/2 cells dramatically repressed Notch activity to induce these markers. This trend also was seen at the transcript level, because Notch activation increased levels of transcript for SMC markers, and this was suppressed by HDAC inhibition (Figure 6B). Finally, TSA enhanced NotchICD/CBF-1 pathway activation (Figure 6C), which is similar to enhanced CBF-1 pathway in human SMCs. These data show that the induction of the SMC contractile phenotype by Notch signaling in human primary SMCs and a murine progenitor cell require HDAC activity but through a mechanism that is independent of canonical CBF-1 signaling.

## Discussion

smcs maintain considerable phenotypic plasticity at the molecular and cellular levels, which is essential for vascular development and is a hallmark of the pathogenesis of vascular diseases. The molecular mechanisms regulating SMC differentiation and maintenance of the contractile SMC phenotype are not completely understood, although multiple pathways regulate SMC phenotypic transitions. Our study is the first to examine the effects of HDAC inhibition on Notch induction of the SMC contractile phenotype. Because inhibition of HDAC activity reduces the level of neointimal lesion formation in vivo,^18,20^ this is an area of interest in considering novel therapies for vascular diseases. Therefore, it is important to understand the relationship of HDAC activity and multiple pathways that regulate SMC phenotype. The HDAC inhibitor TSA caused significant inhibition of the Notch-mediated SMC contractile phenotype, both in human primary aortic SMCs and in a mouse mesenchymal progenitor model. Because TSA is a relatively wide-spectrum HDAC inhibitor, we also used a class I HDAC inhibitor with specificity to HDAC1 and HDAC3 (MS-275). This inhibitor yielded the same activity in regulating SMC markers as TSA. In addition, we utilized a class II inhibitor, which was unable to block Notch activity in inducing SMC markers (data not shown). Therefore, antagonism of Notch signaling is a selective feature of inhibition of class I HDAC. Class I HDAC (HDAC1, HDAC2, and HDAC3) also were the molecules shown to mediate cytokine-stimulated proliferation in rat SMCs.^18^

The effects of HDAC inhibition in human SMCs were gene specific, as the Notch targets of the HRT family were not affected, unlike SMC contractile proteins. This specificity for selective Notch target genes can be explained by our observation that HDAC inhibition does not inhibit canonical CBF-1 activity induced by Notch signaling. In addition, knockdown of CBF-1 activity was not sufficient to completely block the induction of SMC differentiation by Notch. These observations led us to discover alternative signaling pathways activated by Notch signaling. We found that some of the effects of Notch signaling in SMCs can be accounted for by activation of the PI3K/Akt, JNK, and p38 pathways. These pathways are sensitive to HDAC inhibition and are particularly important for increasing calponin1 and SM22α levels. Overall, HDAC inhibition plays a dual role of enhancing CBF-1–mediated Notch signals in SMCs but repressing the Notch-mediated SMC differentiation phenotype. We suggest this dual function is enabled by the activation of multiple pathways downstream of Notch. The canonical CBF-1 and HRT target gene activation is independent of HDAC activity, whereas signaling via PI3K/Akt, JNK, and p38 pathways requires HDAC activity. The requirement of SRF for Notch-mediated SMC differentiation suggests interplay between Notch, HDACs, and SRF in SMC differentiation.

There might be multiple mechanisms of HDAC activity that regulate SMC differentiation. In other studies that examined cytokines that inhibit SMC differentiation (eg, platelet-derived growth factor-BB in rat SMCs^25^), it was shown that suppression of some SMC markers is partially due to HDAC2, HDAC4, and HDAC5 activity in a traditional role of deacetylating histone H4 to silence gene transcription. Likewise, oxidized phospholipid suppression of SM actin in rat SMCs was also associated with recruitment of HDAC2 and HDAC5 and hypoacetylation of histone H4 at the SM actin promoter.^26^ TGFβ, an inducer of SMC differentiation, promotes SM22α transcription, and HDAC inhibition enhances this effect by acetylation of the SM22α promoter.^27^ Although we found that Notch-induced SM22α induction was suppressed by HDAC inhibition, we did observe that the canonical Notch-mediated CBF-1 transcriptional activity was increased, similar to the reported increase in Smad activity. In combination, these data show that the upstream signal activator (eg, platelet-derived growth factor, TGFβ, Notch) and the mechanism of gene target regulation (direct transcriptional activation versus secondary mechanism) will modify the outcome of blocking HDAC activity in SMCs. Class I HDACs also have multiple nonhistone substrates, including transcription factors. This brings up the possibility that both histone and nonhistone targets of HDACs are important to consider in the potential therapeutic use of HDAC inhibitors to regulate SMC differentiation, proliferation, or neointimal lesion formation.

Although substantial evidence shows that Notch signaling regulates SMC differentiation and phenotypic modulation, there are some functional discrepancies, which could be due partly to different experimental systems, species, and phenotypic read-outs. This is further complicated by the fact that Notch interacts with and is regulated by other signaling pathways. For example, we found that the Notch direct target genes of the HRT family have a negative feedback role in Notch-induced SMC differentiation^6^ and also negatively regulate myocardin and TGFβ-mediated SMC differentiation.^5,53,54^ Notch signaling transcriptionally activates SM actin and smooth muscle myosin heavy chain expression through the CBF-1–binding sites in their promoters,^8,36^ and HRTs can repress Notch1ICD induction of SM actin expression by inhibiting Notch1ICD/CBF-1 binding to the SM actin promoter. We also recently found that miR143/145 is a direct transcriptional target of Jagged1/Notch signaling via CBF-1–binding sites in the promoter.^9^ However, the mechanism by which Notch regulates other SMC contractile proteins, such as calponin and SM22α, is still unclear. Although there are CBF-1 consensus–binding sites in the calponin and SM22α promoters, we have not been successful in demonstrating NICD/CBF-1 binding to these promoters with chromatin immunoprecipitation assays in human SMCs (not shown). Therefore, it is likely that although some SMC marker genes are direct transcriptional targets, Notch signaling can induce other contractile genes in an indirect manner, possibility via other signaling pathways or transcriptional mechanisms, including SRF or Smad transcriptional activity.^5^ We found that PI3K/Akt, JNK, and p38 MAPKs are required for Notch-mediated induction of calponin1 and SM22α. However, known direct transcriptional targets such as SM actin were less affected by inhibition of the MAPKs or PI3K pathways. These findings are consistent with our model that different signaling mechanisms act downstream of Notch activation to regulate SMC contractile gene expression.

## References

[1] Yoshida T Owens GK Molecular determinants of vascular smooth muscle cell diversity. Circ Res. 2005;96:280-291 15718508 10.1161/01.RES.0000155951.62152.2e

[2] Owens GK Molecular control of vascular smooth muscle cell differentiation and phenotypic plasticity. Novartis Found Symp. 2007;283:174-191 discussion 191–173, 238–141 18300422 10.1002/9780470319413.ch14

[3] Xie C Zhang J Chen YE MicroRNA and vascular smooth muscle cells. Vitam Horm. 2011;87:321-339 22127249 10.1016/B978-0-12-386015-6.00034-2

[4] Davis-Dusenbery BN Wu C Hata A Micromanaging vascular smooth muscle cell differentiation and phenotypic modulation. Arterioscler Thromb Vasc Biol. 2011;31:2370-2377 22011749 10.1161/ATVBAHA.111.226670 PMC4429757

[5] Tang Y Urs S Boucher J Bernaiche T Venkatesh D Spicer DB Vary CP Liaw L Notch and transforming growth factor-beta (TGFbeta) signaling pathways cooperatively regulate vascular smooth muscle cell differentiation. J Biol Chem. 2010;285:17556-17563 20368328 10.1074/jbc.M109.076414 PMC2878520

[6] Tang Y Urs S Liaw L Hairy-related transcription factors inhibit Notch-induced smooth muscle alpha-actin expression by interfering with Notch intracellular domain/CBF-1 complex interaction with the CBF-1–binding site. Circ Res. 2008;102:661-668 18239137 10.1161/CIRCRESAHA.107.165134 PMC2662732

[7] Doi H Iso T Shiba Y Sato H Yamazaki M Oyama Y Akiyama H Tanaka T Tomita T Arai M Takahashi M Ikeda U Kurabayashi M Notch signaling regulates the differentiation of bone marrow–derived cells into smooth muscle–like cells during arterial lesion formation. Biochem Biophys Res Commun. 2009;381:654-659 19250926 10.1016/j.bbrc.2009.02.116

[8] Noseda M Fu Y Niessen K Wong F Chang L McLean G Karsan A Smooth muscle alpha-actin is a direct target of Notch/CSL. Circ Res. 2006;98:1468-1470 16741155 10.1161/01.RES.0000229683.81357.26

[9] Boucher JM Peterson SM Urs S Zhang C Liaw L The miR-143/145 cluster is a novel transcriptional target of Jagged-1/Notch signaling in vascular smooth muscle cells. J Biol Chem. 2011;286:28312-28321 21685392 10.1074/jbc.M111.221945 PMC3151075

[10] Tang SC Jeng JS Lee MJ Yip PK Notch signaling and CADASIL. Acta Neurol Taiwan. 2009;18:81-90 19673359

[11] Boyer J Crosnier C Driancourt C Raynaud N Gonzales M Hadchouel M Meunier-Rotival M Expression of mutant JAGGED1 alleles in patients with Alagille syndrome. Hum Genet. 2005;116:445-453 15772854 10.1007/s00439-005-1262-7

[12] Guarnaccia C Dhir S Pintar A Pongor S The tetralogy of Fallot-associated G274D mutation impairs folding of the second epidermal growth factor repeat in Jagged-1. FEBS J. 2009;276:6247-6257 19780835 10.1111/j.1742-4658.2009.07333.x

[13] Zhou B Margariti A Zeng L Xu Q Role of histone deacetylases in vascular cell homeostasis and arteriosclerosis. Cardiovasc Res. 2011;90:413-420 21233251 10.1093/cvr/cvr003

[14] Hagelkruys A Sawicka A Rennmayr M Seiser C The biology of HDAC in cancer: the nuclear and epigenetic components. Handb Exp Pharmacol. 2011;206:13-37 21879444 10.1007/978-3-642-21631-2_2

[15] Shabason JE Tofilon PJ Camphausen K Grand rounds at the National Institutes of Health: HDAC inhibitors as radiation modifiers, from bench to clinic. Cell J Mol Med. 2011;15:2735-2744 10.1111/j.1582-4934.2011.01296.x PMC3112261 21362133

[16] Okamoto H Fujioka Y Takahashi A Takahashi T Taniguchi T Ishikawa Y Yokoyama M Trichostatin A, an inhibitor of histone deacetylase, inhibits smooth muscle cell proliferation via induction of p21(WAF1). J Atheroscler Thromb. 2006;13:183-191 16908950 10.5551/jat.13.183

[17] Mathew OP Ranganna K Yatsu FM Butyrate, an HDAC inhibitor, stimulates interplay between different posttranslational modifications of histone H3 and differently alters G1-specific cell cycle proteins in vascular smooth muscle cells. Biomed Pharmacother. 2010;64:733-740 20970954 10.1016/j.biopha.2010.09.017 PMC2997917

[18] Findeisen HM Gizard F Zhao Y Qing H Heywood EB Jones KL Cohn D Bruemmer D Epigenetic regulation of vascular smooth muscle cell proliferation and neointima formation by histone deacetylase inhibition. Arterioscler Thromb Vasc Biol. 2011;31:851-860 21233448 10.1161/ATVBAHA.110.221952 PMC3074344

[19] Song S Kang SW Choi C Trichostatin A enhances proliferation and migration of vascular smooth muscle cells by downregulating thioredoxin 1. Cardiovasc Res. 2010;85:241-249 19633316 10.1093/cvr/cvp263 PMC2791053

[20] Kee HJ Kwon JS Shin S Ahn Y Jeong MH Kook H Trichostatin A prevents neointimal hyperplasia via activation of Krüppel like factor 4. Vascul Pharmacol. 2011;55:127-134 21763782 10.1016/j.vph.2011.07.001

[21] Antos CL McKinsey TA Dreitz M Hollingsworth LM Zhang CL Schreiber K Rindt H Gorczynski RJ Olson EN Dose-dependent blockade to cardiomyocyte hypertrophy by histone deacetylase inhibitors. J Biol Chem. 2003;278:28930-28937 12761226 10.1074/jbc.M303113200

[22] Cardinale JP Sriramula S Pariaut R Guggilam A Mariappan N Elks CM Francis J HDAC inhibition attenuates inflammatory, hypertrophic, and hypertensive responses in spontaneously hypertensive rats. Hypertension. 2010;56:437-444 20679181 10.1161/HYPERTENSIONAHA.110.154567 PMC2931819

[23] Gallo P Latronico MV Gallo P Grimaldi S Borgia F Todaro M Jones P Gallinari P De Francesco R Ciliberto G Steinkuhler C Esposito G Condorelli G Inhibition of class I histone deacetylase with an apicidin derivative prevents cardiac hypertrophy and failure. Cardiovasc Res. 2008;80:416-424 18697792 10.1093/cvr/cvn215

[24] Kong Y Tannous P Lu G Berenji K Rothermel BA Olson EN Hill JA Suppression of class I and II histone deacetylases blunts pressure-overload cardiac hypertrophy. Circulation. 2006;113:2579-2588 16735673 10.1161/CIRCULATIONAHA.106.625467 PMC4105979

[25] Yoshida T Gan Q Shang Y Owens GK Platelet-derived growth factor-BB represses smooth muscle cell marker genes via changes in binding of MKL factors and histone deacetylases to their promoters. Am J Physiol Cell Physiol. 2007;292:C886-C895 16987998 10.1152/ajpcell.00449.2006

[26] Yoshida T Gan Q Owens GK Krüppel-like factor 4, Elk-1, and histone deacetylases cooperatively suppress smooth muscle cell differentiation markers in response to oxidized phospholipids. Am J Physiol Cell Physiol. 2008;295:C1175-C1182 18768922 10.1152/ajpcell.00288.2008 PMC2584997

[27] Qiu P Ritchie RP Gong XQ Hamamori Y Li L Dynamic changes in chromatin acetylation and the expression of histone acetyltransferases and histone deacetylases regulate the SM22alpha transcription in response to Smad3-mediated TGFbeta1 signaling. Biochem Biophys Res Commun. 2006;348:351-358 16876108 10.1016/j.bbrc.2006.07.009

[28] Singh N Trivedi CM Lu M Mullican SE Lazar MA Epstein JA Histone deacetylase 3 regulates smooth muscle differentiation in neural crest cells and development of the cardiac outflow tract. Circ Res. 2011;109:1240-1249 21959220 10.1161/CIRCRESAHA.111.255067 PMC3225257

[29] Adler JT Hottinger DG Kunnimalaiyaan M Chen H Histone deacetylase inhibitors upregulate Notch-1 and inhibit growth in pheochromocytoma cells. Surgery. 2008;144:956-961 discussion 961–952 19041003 10.1016/j.surg.2008.08.027 PMC2638099

[30] Stockhausen MT Sjölund J Manetopoulos C Axelson H Effects of the histone deacetylase inhibitor valproic acid on Notch signalling in human neuroblastoma cells. Br J Cancer. 2005;92:751-759 15685243 10.1038/sj.bjc.6602309 PMC2361888

[31] Greenblatt DY Cayo MA Adler JT Ning L Haymart MR Kunnimalaiyaan M Chen H Valproic acid activates Notch1 signaling and induces apoptosis in medullary thyroid cancer cells. Ann Surg. 2008;247:1036-1040 18520232 10.1097/SLA.0b013e3181758d0e PMC2904809

[32] Greenblatt DY Vaccaro AM Jaskula-Sztul R Ning L Haymart M Kunnimalaiyaan M Chen H Valproic acid activates Notch-1 signaling and regulates the neuroendocrine phenotype in carcinoid cancer cells. Oncologist. 2007;12:942-951 17766653 10.1634/theoncologist.12-8-942

[33] Hautmann MB Madsen CS Owens GK A transforming growth factor beta (TGFbeta) control element drives TGFbeta-induced stimulation of smooth muscle alpha-actin gene expression in concert with two CArG elements. J Biol Chem. 1997;272:10948-10956 9099754 10.1074/jbc.272.16.10948

[34] Minucci S Pelicci PG Histone deacetylase inhibitors and the promise of epigenetic (and more) treatments for cancer. Nat Rev Cancer. 2006;6:38-51 16397526 10.1038/nrc1779

[35] Guo W Shan B Klingsberg RC Qin X Lasky JA Abrogation of TGF-beta1–induced fibroblast-myofibroblast differentiation by histone deacetylase inhibition. Am J Physiol Lung Cell Mol Physiol. 2009;297:L864-L870 19700647 10.1152/ajplung.00128.2009 PMC2777501

[36] Doi H Iso T Sato H Yamazaki M Matsui H Tanaka T Manabe I Arai M Nagai R Kurabayashi M Jagged1-selective Notch signaling induces smooth muscle differentiation via a RBP-Jkappa–dependent pathway. J Biol Chem. 2006;281:28555-28564 16867989 10.1074/jbc.M602749200

[37] Winston JT Koepp DM Zhu C Elledge SJ Harper JW A family of mammalian F-box proteins. Curr Biol. 1999;9:1180-1182 10531037 10.1016/S0960-9822(00)80021-4

[38] Tetzlaff MT Yu W Li M Zhang P Finegold M Mahon K Harper JW Schwartz RJ Elledge SJ Defective cardiovascular development and elevated cyclin E and Notch proteins in mice lacking the Fbw7 F-box protein. Proc Natl Acad Sci U S A. 2004;101:3338-3345 14766969 10.1073/pnas.0307875101 PMC373463

[39] Tsunematsu R Nakayama K Oike Y Nishiyama M Ishida N Hatakeyama S Bessho Y Kageyama R Suda T Nakayama KI Mouse Fbw7/Sel-10/Cdc4 is required for Notch degradation during vascular development. J Biol Chem. 2004;279:9417-9423 14672936 10.1074/jbc.M312337200

[40] Ishikawa Y Onoyama I Nakayama KI Nakayama K Notch-dependent cell cycle arrest and apoptosis in mouse embryonic fibroblasts lacking Fbxw7. Oncogene. 2008;27:6164-6174 18641686 10.1038/onc.2008.216

[41] Guarani V Deflorian G Franco CA Kruger M Phng LK Bentley K Toussaint L Dequiedt F Mostoslavsky R Schmidt MH Zimmermann B Brandes RP Mione M Westphal CH Braun T Zeiher AM Gerhardt H Dimmeler S Potente M Acetylation-dependent regulation of endothelial Notch signalling by the SIRT1 deacetylase. Nature. 2011;473:234-238 21499261 10.1038/nature09917 PMC4598045

[42] Tang Y Yang X Friesel RE Vary CP Liaw L Mechanisms of TGF-β–induced differentiation in human vascular smooth muscle cells. J Vasc Res. 2011;48:485-494 21832838 10.1159/000327776 PMC3169366

[43] Hayashi K Takahashi M Kimura K Nishida W Saga H Sobue K Changes in the balance of phosphoinositide 3-kinase/protein kinase B (Akt) and the mitogen-activated protein kinases (ERK/p38MAPK) determine a phenotype of visceral and vascular smooth muscle cells. J Cell Biol. 1999;145:727-740 10330402 10.1083/jcb.145.4.727 PMC2133182

[44] Wang CC Gurevich I Draznin B Insulin affects vascular smooth muscle cell phenotype and migration via distinct signaling pathways. Diabetes. 2003;52:2562-2569 14514641 10.2337/diabetes.52.10.2562

[45] Lien SC Usami S Chien S Chiu JJ Phosphatidylinositol 3-kinase/Akt pathway is involved in transforming growth factor-beta1–induced phenotypic modulation of 10T1/2 cells to smooth muscle cells. Cell Signal. 2006;18:1270-1278 16310342 10.1016/j.cellsig.2005.10.013

[46] Chen S Crawford M Day RM Briones VR Leader JE Jose PA Lechleider RJ RhoA modulates Smad signaling during transforming growth factor-beta–induced smooth muscle differentiation. J Biol Chem. 2006;281:1765-1770 16317010 10.1074/jbc.M507771200 PMC1831550

[47] Deaton RA Su C Valencia TG Grant SR Transforming growth factor-beta1–induced expression of smooth muscle marker genes involves activation of PKN and p38 MAPK. J Biol Chem. 2005;280:31172-31181 15980430 10.1074/jbc.M504774200

[48] Favata MF Horiuchi KY Manos EJ Daulerio AJ Stradley DA Feeser WS Van Dyk DE Pitts WJ Earl RA Hobbs F Copeland RA Magolda RL Scherle PA Trzaskos JM Identification of a novel inhibitor of mitogen-activated protein kinase kinase. J Biol Chem. 1998;273:18623-18632 9660836 10.1074/jbc.273.29.18623

[49] Bennett BL Sasaki DT Murray BW O'Leary EC Sakata ST Xu W Leisten JC Motiwala A Pierce S Satoh Y Bhagwat SS Manning AM Anderson DW SP600125, an anthrapyrazolone inhibitor of Jun N-terminal kinase. Proc Natl Acad Sci U S A. 2001;98:13681-13686 11717429 10.1073/pnas.251194298 PMC61101

[50] Warrior U Chiou XG Sheets MP Sciotti RJ Parry JM Simmer RL Surber BW Burns DJ Beutel BA Mollison KW Djuric SW Trevillyan JM Development of a p38 kinase binding assay for high throughput screening. J Biomol Screen. 1999;4:129-135 10838421 10.1177/108705719900400306

[51] Du KL Ip HS Li J Chen M Dandre F Yu W Lu MM Owens GK Parmacek MS Myocardin is a critical serum response factor cofactor in the transcriptional program regulating smooth muscle cell differentiation. Mol Cell Biol. 2003;23:2425-2437 12640126 10.1128/MCB.23.7.2425-2437.2003 PMC150745

[52] Li S Wang DZ Wang Z Richardson JA Olson EN The serum response factor coactivator myocardin is required for vascular smooth muscle development. Proc Natl Acad Sci U S A. 2003;100:9366-9370 12867591 10.1073/pnas.1233635100 PMC170924

[53] Doi H Iso T Yamazaki M Akiyama H Kanai H Sato H Kawai-Kowase K Tanaka T Maeno T Okamoto E Arai M Kedes L Kurabayashi M HERP1 inhibits myocardin-induced vascular smooth muscle cell differentiation by interfering with SRF binding to CArG box. Arterioscler Thromb Vasc Biol. 2005;25:2328-2334 16151017 10.1161/01.ATV.0000185829.47163.32

[54] Proweller A Pear WS Parmacek MS Notch signaling represses myocardin-induced smooth muscle cell differentiation. J Biol Chem. 2005;280:8994-9004 15634680 10.1074/jbc.M413316200

